# Vascularized Bone Tissue Formation Induced by Fiber-Reinforced Scaffolds Cultured with Osteoblasts and Endothelial Cells

**DOI:** 10.1155/2013/854917

**Published:** 2013-12-04

**Authors:** Xinhui Liu, Guoping Zhang, Chuanyong Hou, Hua Wang, Yelin Yang, Guoping Guan, Wei Dong, Hongyang Gao, Qingling Feng

**Affiliations:** ^1^Department of Orthopaedics, The Affiliated Jiangning Hospital of Nanjing Medical University, Nanjing 211100, China; ^2^Department of Orthopaedics, The First Hospital of Hebei Medical University, Shijiazhuang 050031, China; ^3^Department of Materials Science and Engineering, Tsinghua University, Beijing 100084, China

## Abstract

The repair of the damaged bone tissue caused by damage or bone disease was still a problem. Current strategies including the use of autografts and allografts have the disadvantages, namely, diseases transmission, tissue availability and donor morbidity. Bone tissue engineering has been developed and regarded as a new way of regenerating bone tissues to repair or substitute damaged or diseased ones. The main limitation in engineering in vitro tissues is the lack of a sufficient blood vessel system, the vascularization. In this paper, a new-typed hydroxyapatite/collagen composite scaffold which was reinforced by chitosan fibers and cultured with osteoblasts and endothelial cells was fabricated. General observation, histological observation, detection of the degree of vascularization, and X-ray examination had been done to learn the effect of vascularized bone repair materials on the regeneration of bone. The results show that new vessel and bone formed using implant cultured with osteoblasts and endothelial cells. Nanofiber-reinforced scaffold cultured with osteoblasts and endothelial cells can induce vascularized bone tissue formation.

## 1. Introduction

It is a problem to repair the damaged bone tissue caused by damage or bone disease. Some current strategies including the use of autografts and allografts have the disadvantages, namely, diseases transmission, tissue availability and donor morbidity. Thus, there is a need to develop the artificial bone substitutes to repair the damaged bone tissue. Bone tissue engineering has been regarded as a new way of regenerating bone tissues to repair or substitute damaged or diseased ones [1].

The main limitation in engineering in vitro tissues is the lack of a sufficient blood vessel system, the vascularization. In vivo, nearly all tissues are supplied with nutrients and oxygen by a highly branched system of larger blood vessels, which are subdivided in the tissue into small capillaries. Some strategies have been developed for the formation of the vessel system in vivo. The first one is based on the endothelial cells and their ability to form new vessels known as neoangiogenesis. Herein prevascularization techniques are compared to approaches in which biomolecules, such as growth factors, cytokines, peptides, and proteins, as well as cells, are applied to generate new vessels. The second strategy is focused on scaffold-based techniques. Naturally derived scaffolds, which contain vessels, are distinguished from synthetically manufactured matrices [2].

Biomaterials are often designed to mimic extracellular matrices (ECM) due to the critical role of the ECM in regulating cell function. Biomaterials with chemical and/or structural similarity to native ECM have been shown to improve cell function [3–5]. An ideal bone tissue engineering graft should have both excellent proosteogenesis and proangiogenesis to rapidly realize the bone regeneration in vivo [6]. The reconstruction of bone defects based on cell-seeded constructs requires a functional microvasculature that meets the metabolic demands of the engineered tissue. Combining the use of cells and materials may open new options for tissue/organ repair and regeneration [7].

In this paper, a new-typed hydroxyapatite/collagen composite scaffold reinforced by chitosan fibers and cultured with osteoblasts and endothelial cells which could support the vascularization of a complex tissue engineered construct for bone [8] has been discussed. Hydroxyapatite and collagen are the main components in natural bone. It has also been proved that nHAC is a very biodegradable and biocompatible material,which can enhance the formation of new bone tissue by increasing osteoblast adhesion, osseointegration, and deposition of calcium-containing minerals on its surface [9–11]. Besides, it has been reported that chitosan can promote adhesion and functional expression of osteoblasts because of its similarity to glycosaminoglycan in structure [12–14]. The biodegradable and biocompatible nanostructured chitosan fibres were added to the scaffold and a porous collagen-based bone scaffold, strengthened with chitosan fibers, with satisfactory porosity properties was prepared. Osteoblasts and endothelial cells have been cultured with the fiber-reinforced scaffolds for the formation of vessels system in vivo.

## 2. Materials and Methods

### 2.1. Reagents and Instruments

They include lymphocyte separation medium (percoll, 1.073 g/mL, Shanghai Huamei Company); DMEM medium (Gibco, USA); heparin, ascorbic acid, dexamethasone, and glycerophosphate (Gibco, USA); trypsin (Sigma Inc., USA); fetal calf serum (Hangzhou Evergreen Company); multilabel immunoassay system (1420, WALLAC, BLCTOR2); Leica Microsystems Heidelberg (GmbH); IVcollagenase (Sigma, USA); 4% paraformaldehyde and 2% glutaraldehyde ECGF (Sigma USA); mouse antihuman factor VIII monoclonal antibody (anti-F VIII-RAg, Boster); multilabel immunoassay system (1420, WALLAC BLCTOR); centrifuge (Heraeus, Germany); inverted microscope (Chongguang Chongqing); immunofluorescence microscopy (Leica, Germany).

### 2.2. Materials

Chitosan fibres (80% deacetylated; diameter, 12.5 *μ*m; tensile strength, 550 MPa) were obtained from Donghua University, China; PLLA was purchased from the Shandong Medical Appliance Factory, China, while type I collagen (at a concentration of 1%), dioxane and other chemicals were purchased from the Beijing Chemical Company Ltd, China; osteoblasts and endothelial cells of rabbit. New Zealand rabbits (8–10 weeks old, about 2 kg, without distinguishing gender). All animals were from a closed colony.

### 2.3. Methods

#### 2.3.1. Preparation of Composite Scaffold Reinforced by Chitosan Fibers

The scaffold was prepared based on the study of Li et al. [5, 6]. First, type I collagen was dissolved in acetic acid to give a solution with a concentration of 0.0015 g mL^−1^. Then, a solution containing PO_4_^3−^ was added to the collagen solution until the content ratio of PO_4_^3−^/collagen reached 0.05 mol g^−1^. Next, a solution containing Ca^2+^ was added to the previous solution until the molar ratio of Ca^2+^/PO_4_^3−^ reached 1.66, followed by the addition of NaOH solution until the pH of the system (determined with a pH meter) was about 7. Finally, after standing for 2 d, the resulting solution was centrifuged and the deposit was lyophilized after rinsing (three times) with distilled water. Subsequently, the lyophilized deposit was ground into a fine powder.

PLLA, with a weight-average molecular weight of 105 g mol^−1^, was dissolved in dioxane (pore-forming agent) at a concentration of 0.08 g mL^−1^. Then, nHAC powder was added to the solution until the weights of nHAC and PLLA were equal. Finally, the prepared liquid was dispersed ultrasonically for 30 min and the system lyophilized for 12 h.

The chitosan fibres were gradually added to the nHAC/PLLA solution, while being stirred with a magnetic stirrer. Then, the liquid was dispersed ultrasonically for 45 min and finally the system was lyophilized for 12 h.

The composites were cut into the size of 15 mm × 15 mm × 5 mm, sterilized by Co_60_.

#### 2.3.2. Vascularization of Tissue Engineering Bone In Vitro

The osteoblasts and endothelial cells were planted in the four surfaces of the prepared nanomaterials. 4–6 hours after the inoculation, the serum-free medium was added to maintain cells nutrition in vitro and the nanoscaled vascularized tissue engineering bone was prepared and retained.

#### 2.3.3. Rabbit Radius Bone Defect Model

30 New Zealand white rabbits (male or female) have been prepared. Sodium pentobarbital (35 mg/kg) was used for intravenous anesthesia. The rabbits were fixed in supine position. The right forelimbs of rabbits were disinfected and then cut along the anterolateral part longitudinally. Periosteum was cut apart and the 1/3 part of radial bone in the upper was exposed. Then radial bone was sawn into sections about 15 mm to make bone defect, and then periosteum was completely resected. Finally, rabbit radius bone defect model was manufactured, and the male rabbits and female rabbis were raised separately.

#### 2.3.4. Surgical Repair of Bone Defect Animal Model

Vascularized tissue engineering bone was implanted in the rabbit radius bone defect. Use alcohol on the wound for local disinfection every day within a week after the operation and inject 2 mL lincomycin to every rabbit.

#### 2.3.5. Obtaining Animal Specimens after Ink Perfusion

The animals were killed, respectively, after 4, 8, and 12 weeks. The animals were under general anesthesia before obtaining specimens. Cut apart the rabbit, expose the heart, and inject ink-formaldehyde mixture (ink : formaldehyde = 6 : 1) about 10 mL into the left ventricle, until observing that the skin and mucous membranes of rabbit's face turn black. Obtain the specimens after 2 h. The specimens were placed into 10% formaldehyde solution for the latter histological examination.

### 2.4. Observation and Testing Indicators

#### 2.4.1. General Observation

After surgery, the animal's diet, activity and other systemic conditions, local wound healing, and limb swelling were observed. Angiogenesis on the specimen surface and the materials degradation were observed after obtaining the specimens.

#### 2.4.2. Histological Observation

Specimens had been decalcified for 15 days after being fixed with formaldehyde. Embed specimens in paraffin and slice with a slicing machine at the thickness of 20 *μ*m for the observation of angiogenesis and osteogenesis.

#### 2.4.3. Detection of the Degree of Vascularization

Three horizons of each slice from the ink perfusion specimens which were obtained at week 4, week 8, and week 12 were randomly selected. Ink image is collected and entered into a computer image analysis system; the ink color percentages of area occupied by the image were calculated to obtain the degree of vascularization.

#### 2.4.4. X-Ray Examination

Take photos with the Philips 500 mAX ray machine in a small focus and shoot the film, at weeks 4, 8, and 12 after surgery. The exposure conditions were unified, at 45 Kv, 3–5 mAs. Callus formation and bone healing of bone defects area were observed.

## 3. Results

### 3.1. General Observation

The diet and activity of rabbits are normal after the surgery. The limbs were mildly swelling and then detumescence occured after 1 week. Except the four rabbits that died of unexplained diarrhea accidentally, all the other rabbits were surviving until the period required for the experiment. In addition, all the wounds of the rabbit were healing and the sutures were bit away by the rabbits themselves.

Four weeks after the surgery, all plants kept steady without position changing. There were fibrous connective tissue connecting together between the materials and radius bone. Complete fibrous tissue capsule formed on the surface of the implants, while the combination between the capsule and implants was loosened. A large number of blood vessels dyed black ink can be seen. Eight weeks after the surgery, the combination between the radius bone and the implants was stable, as well as the combination between capsule and implants. The area of blood vessels dyed black ink was more than 4 weeks ago. 12 weeks after the surgery, the combination between the radius bone and the implants became firm. The bounds between bone and implants were indistinct.

### 3.2. Histological Observation

8, 8, and 12 specimens were obtained at week 4, week 8, and week 12. The ink perfusion tissue sections and conventional tissue sections occupied half, respectively, among the specimens obtained at different periods.

**Figure 1:**
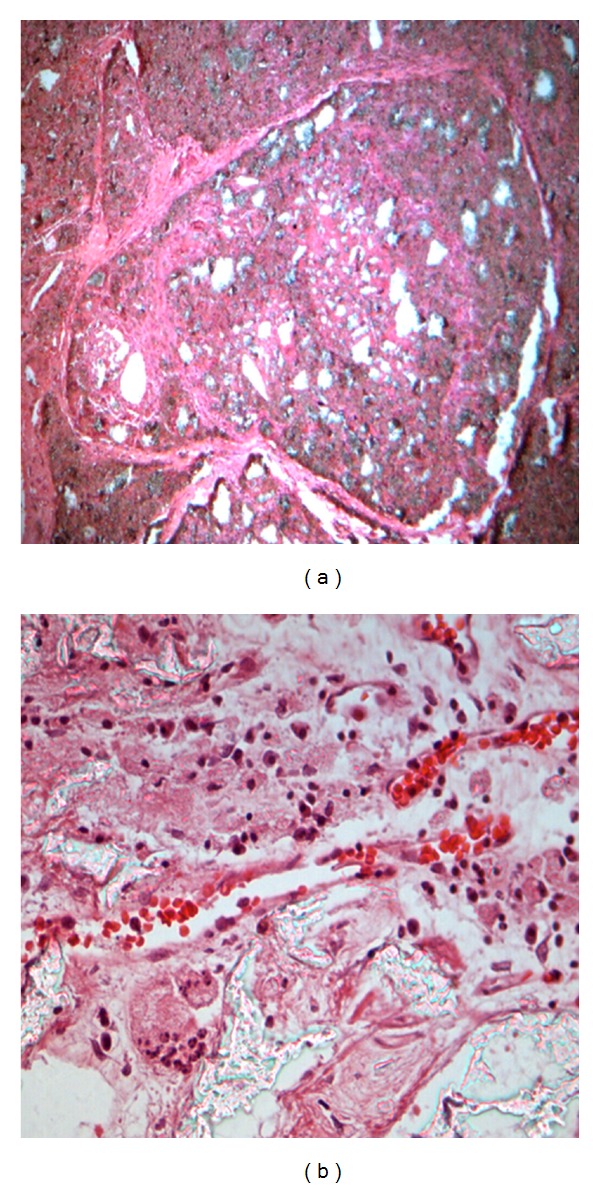
Different tissue sections with different enlargement factors at week 4 ((a) 100 times enlarged ink perfusion tissue sections; (B) 400 times enlarged conventional tissue sections).

At week 4, it could be observed that fibrous connective tissue had grown inside the implant materials and separated the materials in the ink perfusion specimens. Most of the area of the ink perfusion specimens appeared in ink color. It was demonstrated that the new vessel had formed and had been dyed. Only the center of the specimens had not been dyed. It was demonstrated that new vessel had not formed at this part. A large moment of fibrous callus and osteoid had formed in the dyed part. The original shape of the implant materials had gradually disappeared and just only remained in the center of the material. Osteogenesis phenomenon had not been seen at week 4 (Figure 1(a)). New vessel could be seen in the conventional tissue sections, as well as the endothelial cells of the periphery vessel wall and a large number of erythrocytes in the blood vessel. A mass of fibroblasts, a number of lymphocytes, plasma cells, and neutrophils can be observed as well as osteoclasts and osteoblasts. Prototype materials had disappeared and materials had been replaced by fibrous osteotylus. A small amount of osteoid formed. New blood vessel could not be seen in the center of the conventional tissue sections as well as the bone repair phenomena. The original shape of the implant materials had been clear in the center of the conventional tissue sections (Figure 1(b)).

**Figure 2:**
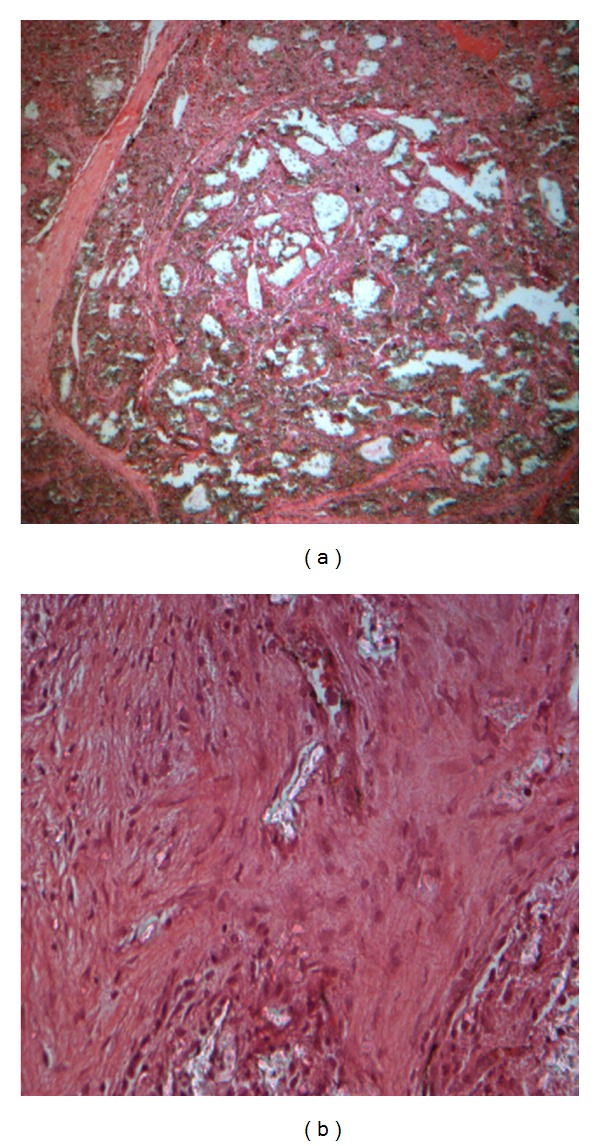
Different tissue sections with different enlargement factors at week 8 ((a) 100 times enlarged ink perfusion tissue sections; (b) 400 times enlarged conventional tissue sections).

At week 8, it could be observed that more fibrous connective tissue had grown into the materials. New vessels had formed, as the center of the specimens turned black. Osteoid could be seen in most part of the ink perfusion pecimens (Figure 2(a)). Fibrous callus could be seen in the center of the conventional tissue sections. Osteoid could also be seen in the most part of the materials. A small amount of bone callus could be seen around the materials which had been divided. Monocytes, plasma cells, and lymphocytes had disappeared and been replaced by a number of fibroblasts, osteoblasts, and osteoclasts (Figure 2(b)).

**Figure 3:**
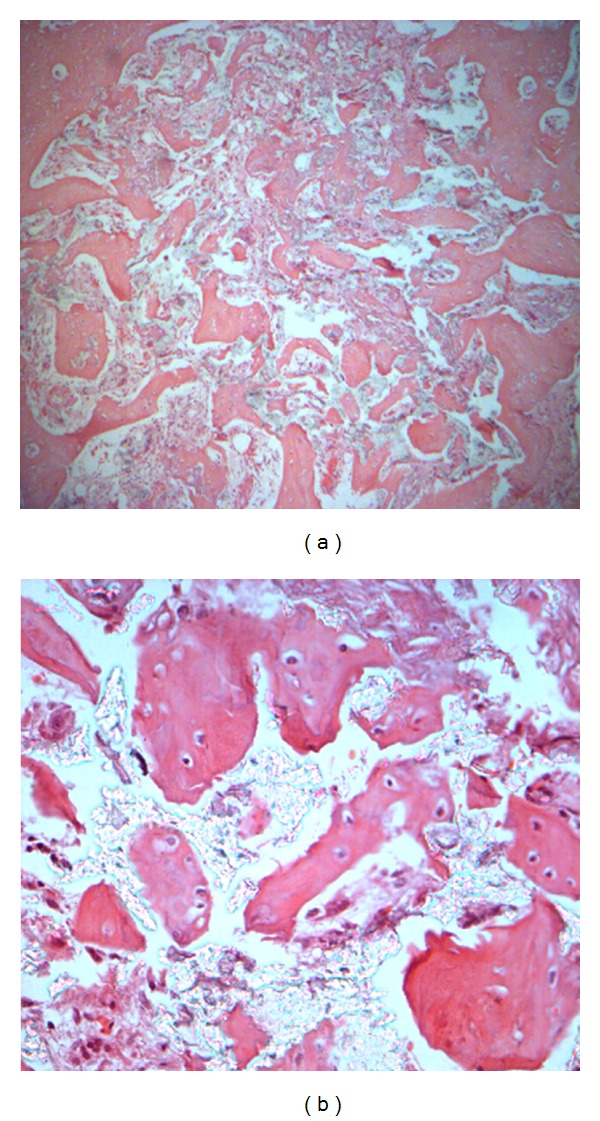
Different tissue sections with different enlargement factors at week 12 ((a) 100 times enlarged ink perfusion tissue sections; (b) 400 times enlarged conventional tissue sections).

At week 12, the ink color of the ink perfusion specimens had turned light and had been replaced by the red color of a large amount of osteoids, which formed recently in the ink perfusion group. All materials were already fully vascularized. Scattered new bone osteotylus, osteoid and a small amount of fibrous osteotylus existed at the same time. The newly formed bone callus gradually joined together to form a larger bone callus (Figure 3(a)). These phenomena could be seen even more clearly in the conventional slice. The bone lacunae and osteocyte could be observed in the bone osteotylus. Osteoclasts could be seen around the newly formed the bone osteotylus. It meant that new bone was forming when bone repaired and remodeled (Figure 3(b)).

### 3.3. Detection of the Degree of Vascularization

13 ink perfusion specimens were obtained, respectively, in this experiment (4 specimens at week 4, 8 specimens at week 8, 5 specimens at week 12). Vascularized area gradually increased and then further increased to 100 percentages at week 12 (Table 1).

### 3.4. X-Ray Examination

**Figure 4:**
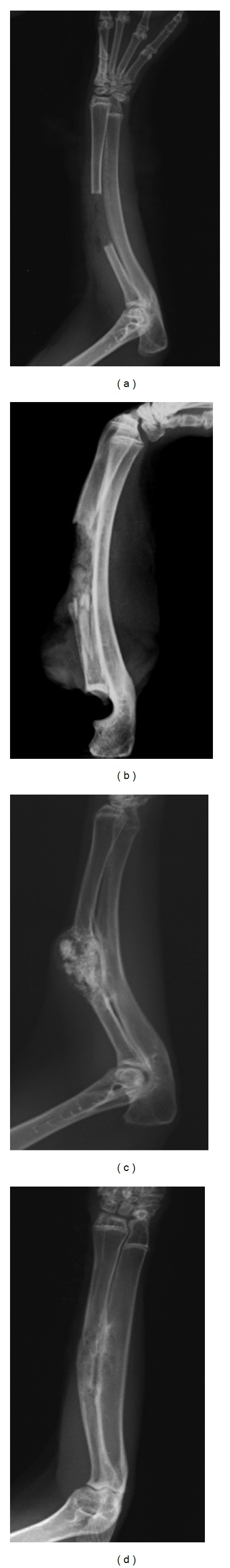
X-ray image of the rabbits' radial bones taken at different periods. (a) X-ray image after making bone defect about 15 mm; (b) X-ray image at week 4; (c) X-ray image at week 8; (d) X-ray image at week 12.

X-ray examination was done as soon as the surgery was finished. It was successful to make bone defect about 15 mm at the upper one-third radial bones (Figure 4(a)).

The implants develop with low density. There were some high-density developments in the bone defect area. It meant that new bone had formed at week 4 (Figure 4(b)).

The high-density development caused by bone formation at week 8 turned larger than that at week 4 (Figure 4(c)). It could be seen that new bone formed in bone defect area and had connected the farther and the nearer side. The bone marrow cavity had formed in the newly formed bone at week 12 (Figure 4(d)).

## 4. Discussion

Bone grafting is the preferred method to treat bone defects [15, 16]. Vascularization of the graft determines whether the bone grafting is successful. In vivo nearly all tissues are supplied with nutrients and oxygen by a highly branched system of larger blood vessels, which are subdivided in the tissue into small capillaries. A good environment ensures the growth, differentiation, and proliferation of osteoblasts [4, 17, 18]. In the process of bone healing, mineral deposition is the main mechanism of blood flow control, rather than the physiological metabolic changes of periosteum. All in all, vascularization and osteogenic cells are closely related.

Current strategies for bone grafts including the use of autografts, allografts, and synthetic grafts have drawbacks. Bone tissue engineering provides a new way for regenerating bone tissues to repair or substitute damaged or diseased ones [19–24]. While the main limitation in engineering in vitro tissues is the lack of a sufficient blood vessel system, the vascularization, as well as the bone tissue engineering. Successful cell-based tissue engineering requires a rapid and thorough vascularization in order to ensure long-term implant survival and tissue integration [25, 26]. To rebuild the blood supply at the same time of building bone tissue engineering Is a main problem. The lack of adequate nutrition may have bad effect on the proliferation, differentiation, and secretion of seed cells in vitro environment and even cause the death of the seed cells, after the implantation of artificial bone. Thus, establishing blood transfer system as soon as possible can retain the osteogenesis advantage of artificial bone and bring more osteoblasts factor which can further promote the rapid regeneration of bone tissue.

The reconstruction of bone defects based on cell-seeded constructs requires a functional microvasculature that meets the metabolic demands of the engineered tissue. So, strategies that augment neovascularization need to be identified [27]. We propose an in vitro strategy consisting of the simultaneous culture of osteoblasts and endothelial cells on a collagen-based scaffold for the formation of prevascular structures, with the final aim of accelerating the establishment of a vascular bed in the implanted construct and supports the reformation of bone. In this paper, the vascularized tissue engineering bone cultured with vascular osteoblasts and endothelial cells was used as implant to repair the rabbit's radius bone defect. The ink perfusion method was used to study the angiogenesis and the relationship between angiogenesis and oteogenesis.

Ink was perfused into the left ventricle of rabbit. The tissue can only be dyed black at the existence of vessel. The ink color could be seen where new tissue formed, because there was no vessel in the materials we implant previously. The black area of ink perfusion specimens increased gradually as time passed. It meant that it was possible to vascularize the artificial bone cultured with vascular endothelial cells and that vascular endothelial cells were ideal seed cells for vascularization [28–30].

The size of the bone defect is directly related to the objectivity and reliability of the experiment. Hollinger and Kleinschmidt [31] proposed the concept of critical size of bone defects. Critical size of bone defects is the size that the bone defect cannot be self-healing without implants in experimental animals. The size of rabbit radius bone defect was based on this principle. It has been demonstrated that the rabbits can repair themselves with the size of bone defect which is less than 15 mm. Rabbit radius bone defect modeling was used in this experiment. Vascularized tissue engineering bone was implanted in this rabbit radius bone defect. Histological observation and X-ray examination were used to demonstrate the good ability to repair bone. The result showed that the HA/collagen composites were ideal extracellular matrix material and had a good biocompatibility in vivo. HA/collagen composites were promising to be an ideal bone substitute.

After scaffold implantation, the growth of capillaries into the porous construct may be too slow to provide adequate nutrients to the cells in the scaffold interior and this inhibits tissue formation in the scaffold core. It was found that the cell differentiation did not happen inside the tissue engineering bone constructed by implanted seed cells on the surface of materials. Vascularization and osteogenesis of the implant may be accelerated by using three-dimensional culture technology and culturing the seed cells inside the materials. Supporting nutrient and maintaining physiological function of tissue engineering bone in vivo were still a main problem. In short, the vascularization of tissue engineering bone will always be a task we should continue.

## 5. Conclusions

The present study demonstrates that the nanofiber reinforced HA/collagen composites cultured with osteoblasts and endothelial cells can induce vascularized bone tissue formation. The composites have a good biocompatibility in vivo and are promising to be an ideal bone substitute, which can be implanted as bone graft with inducing visualization and osteogenesis. However, because of the poor vascularization inside of scaffolds, further studies should investigate new strategies that can accelerate and improve the vascularization process in bone, where vascularization is a prerequisite to achieving optimal regeneration.

## Figures and Tables

**Table 1:** The degree of vascularization at different period.

4 w	8 w	12 w
85.13 ± 0.21	96.77 ± 0.24	100
